# Parent-Reported Symptoms of Acute Otitis Media during the First Year of Life: What Is beneath the Surface?

**DOI:** 10.1371/journal.pone.0121572

**Published:** 2015-04-07

**Authors:** Alexandre C. Fortanier, Roderick P. Venekamp, Marieke L. A. de Hoog, Cuno S. P. M. Uiterwaal, Anne C. van der Gugten, Cornelis K. van der Ent, Arno W. Hoes, Anne G. M. Schilder

**Affiliations:** 1 Julius Center for Health Sciences and Primary Care, University Medical Center Utrecht, Utrecht, The Netherlands; 2 Department of Otorhinolaryngology, University Medical Center Utrecht, Utrecht, The Netherlands; 3 Department of Pediatric Pulmonology, Wilhelmina Children's Hospital, University Medical Center Utrecht, Utrecht, The Netherlands; 4 Ear Institute, University College London, London, United Kingdom; Rockefeller University, UNITED STATES

## Abstract

**Background:**

Most estimates of the incidence of acute otitis media (AOM) are based on general practitioner (GP) or pediatrician diagnoses. It is likely that these figures underestimate the community incidence of AOM since parents do not visit their doctor every time their child suffers from acute ear symptoms. The impact of these symptom episodes may be substantial since they affect the child’s quality of life and parents’ productivity.

**Methods:**

To determine AOM symptoms in the community, we measured parent-reported AOM symptoms daily for 12 consecutive months in 1,260 children participating in a prospective birth cohort in the Netherlands. The mean age of these children was at study enrollment 0.9 months (standard deviation 0.6). A parent-reported AOM symptom episode was defined as fever (temperature 38˚C or above) plus at least one of the following symptoms: ear pain and ear discharge. These febrile AOM symptom episodes were linked to GP-consultations and diagnoses in the GP electronic health records.

**Results:**

With an estimated 624 parent-reported symptom episodes per 1,000 child-years (95% CI: 577 to 674) incidence of febrile AOM symptoms during the child’s first year is high. The GP was consulted in half of these symptom episodes and AOM was diagnosed in 49% of these consultations.

**Conclusions and Relevance:**

The incidence of febrile AOM symptoms in the first year of life is high in Dutch children and leads to a GP-consultation in only half of the cases. This suggests that AOM symptomatology in the community is underestimated when focusing on GP-diagnosed AOM episodes alone, since a considerable proportion of febrile AOM symptom episodes are treated symptomatically by parents at home and do not come to the attention of the GP. Having data on community AOM symptomatology available for each country is important when the potential impact of preventive and therapeutic interventions for AOM are studied.

## Introduction

Acute otitis media (AOM) is one of the most common childhood respiratory infections with most episodes occurring during early infancy [1]. Publications on the epidemiology of AOM often present physician diagnoses of AOM as a proxy for the community incidence of AOM [2]. However, incidence estimates based on physician diagnoses are likely to underestimate the incidence of AOM since parents do not consult their physician every time their child develops symptoms of AOM [3]. At the same time such symptom episodes may have considerable impact on the child’s and family’s quality of life and lead to substantial societal costs [3–6].

Beside a recent study [7], the few studies so far that have used parental reporting to estimate AOM symptoms in the community were retrospective (introducing the risk of recall bias) and were not able to link the parent-reported episodes to health record information [3–6,8,9]. In addition, most were performed before the implementation of pneumococcal conjugate vaccination. The introduction of these vaccines in childhood may have changed the causative pathogens involved in AOM and subsequently the incidence and pattern of AOM [10,11].

Previous studies on AOM have shown that parent-reported symptoms may have important diagnostic value in particular ear pain, fever and ear discharge, [12–15] although some argue that symptoms or symptom-based scores cannot predict AOM [16].

Recognizing that accurate information on the incidence of AOM within the community is essential to provide valid estimates on costs and potential benefits of interventions to prevent or treat AOM, we monitored AOM symptomatology within a large prospective birth cohort study in the Netherlands and linked these data to general practitioner (GP) health records. In the Netherlands the GP is the gatekeeper to the Dutch healthcare system both during and outside of office hours and children need a specialist referral from a GP before visiting secondary or tertiary care.

## Methods

Data on AOM-related symptoms, GP-consultations and GP-diagnoses were retrieved from 1,260 children participating in a Dutch prospective birth cohort study (WHISTLER) from 2008 and onwards [17]. The WHISTLER project was approved by the medical ethical committee of the University Medical Center Utrecht, The Netherlands (project approval number 01/176). After obtaining written informed consent from both parents, children were enrolled within the first 2 months of life. Parents received face-to-face instructions by a member of the study team to complete a monthly questionnaire including questions on their child’s respiratory symptoms for each day of the months for 12 consecutive months. Parents’ compliance was optimized by telephone and written reminders.

A parent-reported AOM symptom episode was defined as a diary entry of fever (temperature > 38°C) in combination with either ear pain and/or ear discharge. An episode was counted if ear pain and/or ear discharge occurred within 24 hours after the onset of fever, irrespective of persistence of fever throughout the episode. A new episode was recorded after a symptom free interval of 7 days [9]. Using the calendar dates on the questionnaires, parent-reported AOM symptom episodes were linked to GP-consultations and AOM diagnoses as recorded in the GP electronic health records. GP-diagnoses were coded according to the International Classification of Primary Care (ICPC) coding system [17]. Diagnostic codes of main interest included AOM (ICPC H71), otitis media-related diagnoses [e.g. earache (H01), ear discharge (H04), otitis media with effusion (H72), chronic otitis media (H74)], acute upper respiratory tract infection (URTI) (R74), fever (A03) and coughing (R05). For completeness, other GP-diagnoses were also retrieved.

Pneumococcal conjugate vaccination has been included in the Dutch National Immunization Program since April 2006 for all newborns. In March 2011 the 7-valent vaccine (CRM-PCV7) was replaced by a 10-valent vaccine (PD-PCV10). Although the individual vaccination status within the cohort under study is not known, the overall percentage of children fully vaccinated (4 doses at age 2, 3, 4 and 11 months) is 95.5% for the municipality of Utrecht [18].

Incidence of febrile AOM symptoms in the community per 1,000 child-years was calculated by dividing the number of parent-reported AOM symptom episodes by the total number of child-years. We calculated the proportion of parent-reported AOM symptom episodes that led to a GP-consultation by dividing the number of GP-consultations during the parent-reported AOM symptom episodes by the total number of parent-reported AOM symptom episodes. The proportion of GP-consultations that led to an AOM diagnosis was calculated by dividing the number of GP-diagnoses of AOM (H71) at time of GP-consultations by the total number of GP-consultations during parent-reported AOM symptom episodes. For the other diagnoses of interest (otitis media-related diagnoses, URTI, fever, coughing) the same procedure was followed. In sensitivity analyses, we explored whether our main findings would change when stratifying our parent-reported AOM symptom episodes by three different symptom combinations: fever with ear pain, fever with ear discharge, fever with ear pain and ear discharge. In further sensitivity analyzes, we explored whether our results would change if we only included: (a) children whose parents completed all 12 questionnaires and (b) febrile AOM symptom episodes in which the onset of both fever and ear-symptoms were reported on the same day. All statistical analyses were performed with SPSS version 20.0 (SPSS Inc, Chicago, ILL, USA) and OpenEpi: Open Source Epidemiologic Statistics for Public Health (version 3.03, updated September 2014).

## Results

Table 1 summarizes the characteristics of the 1,260 included children with a total duration of follow-up of 1,029 child-years. 397 children (32%) experienced at least one parent-reported AOM symptom episode in their first year of life. A total of 642 febrile AOM symptom episodes were reported (mean number per child: 1.6, standard deviation (SD): 1.0) with a median duration of 4.0 days; 90% resolved within 16 days. In 484 of the 642 (75%) parent-reported AOM symptom episodes children had fever and ear pain, in 63 (10%) fever and ear discharge and in 95 (15%) fever with both ear pain and ear discharge. The mean age of onset of parent-reported AOM symptom episodes was 9.0 (SD: 2.5) months for children with single febrile AOM symptom episodes (246 children) and 7.1 months (SD: 2.2) for those with multiple episodes (151 children). The incidence of febrile AOM symptoms in the community was 624 episodes per 1,000 child-years (95% CI: 577 to 674). With 56 versus 586 episodes, the number of episodes was much lower in the first 6 months of life than in the second half of the first year of life, reflecting an incidence of 118 versus 1,054 episodes per 1,000 child-years (given the available child-years of 473 and 556 respectively).

**Table 1:** Characteristics of the 1,260 included children.

Total number of included children (%)	1,260 (100)
Boys (%)	609 (48.3)
Mean age at start study, months (SD)	0.9 (0.6)
Mean number of questionnaires returned per child (SD)	9.8 (3.4)
Number of children with 12 completed questionnaires (%)	680 (54.0)
Total duration of follow-up (child-years)	1,029
*In 0–5 months of life (child-years)*	473
*In 6–12 months of life (child-years)* *	556
Median age at first parent-reported AOM episode, months (range)	8.0 (1 to 13)
Children with one or more parent-reported AOM symptom episodes (%):	
*1 episode*	*246 (19*.*5)*
*2 episodes*	*95 (7*.*5)*
*3 episodes*	*30 (2*.*4)*
*4 episodes*	*18 (1*.*4)*
*≥5 episodes*	*8 (0*.*6)*
Total	397 (31.5)

*Children completed up to 12 questionnaires. In some cases also at the age of 13 months a questionnaire was returned, depending on age at start study; SD, standard deviation; child-years being the number of available follow-up years; %, percentage.

For 51% (326 of 642 episodes) of parent-reported AOM symptom episodes the GP was consulted. Compared with all AOM symptom episodes (n = 642), the median duration of symptom episodes for which a GP was consulted (n = 326) was somewhat longer (4.0 days [IQR 7.0] versus 6.0 days [IQR 7.0] respectively; Table 2).

**Table 2:** Characteristics of febrile AOM symptom episodes stratified by GP-consultation and GP-diagnosis of AOM.

	All AOM symptom episodes	AOM symptom episodes for which the GP was consulted	AOM symptom episodes for which the GP was consulted and in which AOM was diagnosed
Number of episodes	642	326	142
Number of children	397	228	114
Boys (%)	201 (51)	113 (50)	55 (48)
Median duration of symptom episode, days (IQR)	4.0 (7.0)	6.0 (7.0)	6.0 (7.3)
Mean number of episodes per child (SD)	1.6 (1.0)	1.6 (1.0)	1.7 (1.1)
Number of episodes in 0–5 months of life (%)	56 (9)	30	15
Median duration of symptom episode, days (IQR)	4.0 (4.8)	5.0 (8.0)	5.0 (6.0)
Number of episodes in 6–12 months of life (%)	586 (91)	296	127
Median duration of symptom episode, days (IQR)	4.0 (7.0)	6.0 (7.0)	6.0 (8.0)

IQR, interquartile range; SD, standard deviation; %, percentage.

In 287 of the 326 (88%) AOM symptom episodes that led to a GP-consultation, a diagnosis was entered in the GP health records; a GP-diagnosis of AOM was recorded in 142 (49%) and a diagnosis of URTI, otitis media-related diagnoses (earache, ear discharge, otitis media with effusion and chronic otitis media), fever and cough was recorded in 44 (15%), 20 (7%), 14 (5%) and 7 (2%) of these consultations, respectively. The most common other GP-diagnoses were acute bronchiolitis/bronchitis (7), gastro-enteritis (6), other viral disease (5), varicella (4) and atopic eczema/dermatitis (4) (see S1 Table Total number of GP-consultations and GP-diagnoses during a parent-reported AOM symptom episode, for full list).

In children younger than 6 months the GP was consulted in 30 of 56 (54%) parent-reported AOM symptom episodes and in 15 of these consultations the GP diagnosed AOM. In children aged 6–12 months the GP was consulted in 296 of 586 (51%) parent-reported AOM episodes and in 127 of these consultations the GP diagnosed AOM (Table 2).

Table 3 summarizes the proportion of parent-reported AOM symptom episodes that led to a GP-consultation and a subsequent GP-diagnosis of AOM, stratified by the three different symptom combinations. Findings of these stratified analyses did not substantially differ from our main analyses in which all combinations of symptoms were included (Table 3).

**Table 3:** Characteristics of all febrile AOM symptom episodes stratified by symptoms.

Episodes with fever plus	ear pain (%)	ear discharge (%)	ear pain and ear discharge (%)	Total number of AOM symptom episodes^ (%)
Total number of episodes	484	63	95	**642**
Number of GP-consultations	243 (50)	27 (43)	56 (59)	326 (51)
Number diagnosis entered in GP health records	31	2	6	39
GP-diagnosis available*:	212	25	50	287
Acute otitis media	104 (49)	11 (44)	27 (54)	**142 (49)**
Upper respiratory tract infection	34 (16)	3 (12)	7 (14)	44 (15)
Otitis media-related^#^	10 (5)	4 (16)	6 (12)	20 (7)
Fever	10 (5)	2 (8)	2 (4)	14 (5)
Cough	6 (3)	1 (4)	0 (0.0)	7 (2)

* ICPC code entered in the GP electronic health record;

^ Parent-reported AOM symptom episode defined as fever in combination with either ear pain and/or ear discharge;

# Otitis media-related diagnoses include earache, ear discharge, otitis media with effusion and chronic otitis media; %, percentage.

Sensitivity analysis including only 680 children (54%) whose parents completed all 12 questionnaires, showed a similar incidence of AOM symptoms in the community as compared to the overall study population (634 versus 624 per 1,000 child-years; p = 0.8). Further sensitivity analysis including only parent-reported AOM symptom episodes with fever and onset of ear-symptoms occurring on the same day, showed an incidence of AOM symptoms in the community of 561 per 1,000 child-years (95% CI: 516 to 608).

## Discussion

Our study shows that the incidence of febrile AOM symptoms in the community is high during children’s first year of life, in particular in the second half of that year, and parents consult their GP in only half of the febrile AOM symptom episodes. To the best of our knowledge, this is the first prospective birth cohort studying the incidence of parent-reported AOM symptom episodes after the widespread implementation of vaccination against *S*. *pneumoniae*.

Compared to a recent survey among parents of children between 0–4 years conducted in seven European countries, we found a higher proportion of children with one or more parent-reported AOM symptom episodes per year, i.e. 32% versus 14–21% [3]. This is likely due to our population being younger (first year of life versus 0–4 years). We found that a substantial proportion of parent-reported AOM episodes did not led to a GP-consultation. These findings are in agreement with data from a recent prospective study conducted in five European countries suggesting that an additional 30% of AOM episodes may be missed when focusing only on data from medical records (110 episodes/1,000 additional on top of 256/1,000 child-years) [7].

Some aspects of our study need further consideration. First, the proportion of parents visiting their GP in case of AOM symptoms was relatively low. In the Netherlands, a watchful waiting strategy with supportive care (analgesics) for the first 3 days has been advocated for decades in children with AOM [19] and the antibiotic prescription rate is relatively low in this condition [20,21]. Dutch parents might therefore perceive a higher threshold to seek medical attention compared with most other Western countries [3,20]. Second, we used fever as the entry criterion for defining parent-reported AOM symptom episodes since we wanted to capture episodes of acute febrile illness and chose ear pain and/or ear drainage as symptoms of important diagnostic value in AOM [12–15]. Our study did not capture afebrile episodes of ear pain and/or drainage, which contribute to an estimated 20% of children diagnosed with AOM in primary care [22] nor did we capture URTI episodes with middle ear involvement presenting without specific ear symptoms as may be common in young children. Third, missing questionnaires may have led to an underestimation of the incidence of AOM symptoms episodes in the community. Since an average of ten questionnaires were returned per child and incidences were similar in children whose parents completed all twelve questionnaires, this is unlikely to have influenced our findings. Fourth, we defined a GP-diagnosis of AOM as an ICPC code H71 recorded in the GP health records. Variation in AOM coding between GPs and misclassification of AOM cannot be ruled out, in particular in this young age group where otoscopic confirmation is known to be difficult [23]. Furthermore, a GP-diagnostic code was not available for 39 of the 326 (12%) GP-consultations. Although this may have led to a slight over- or underestimation of the proportion of AOM diagnoses during GP-consultations, we feel that it is unlikely these missing data have had a major influence on our findings.

## Conclusions and Relevance

The incidence of febrile AOM symptoms in the first year of life is high in Dutch children and leads to a GP-consultation in only half of the cases. This suggests that AOM symptomatology in the community is underestimated when focusing on GP-diagnosed AOM episodes alone, since a considerable proportion of febrile AOM symptom episodes are treated symptomatically by parents at home and do not come to the attention of the GP. Having data on community AOM symptomatology available for each country is important when the potential impact of preventive and therapeutic interventions for AOM are studied.

## Supporting Information

S1 Table Total number of GP-consultations and GP-diagnoses during a parent-reported AOM symptom episode. *ICPC code entered in the GP electronic health record; ^Otitis media-related diagnoses include earache, ear discharge, otitis media with effusion and chronic otitis media; %, percentage.
(DOCX)
