# Supplementary material for: Parent-Reported Symptoms of Acute Otitis Media during the First Year of Life: What Is beneath the Surface?
Source: PLoS One. 2015 Apr 7;10(4):e0121572. doi: 10.1371/journal.pone.0121572 (PMC4388588; doi:10.1371/journal.pone.0121572)
Supplement: S1 Table — *ICPC code entered in the GP electronic health record; ^Otitis media-related diagnoses include earache, ear discharge, otitis media with effusion and chronic otitis media; %, percentage. (DOCX) [file pone.0121572.s001.docx]

|  | **All children (%)** |
| --- | --- |
| Total number of episodes | 642 |
| GP-consultations | 326 (51) |
| No GP-diagnosis (ICPC code) entered in GP health records | 39 (12) |
| GP-diagnosis available*: | 287 |
|  |  |
| Acute otitis media | 142 (49) |
| Upper respiratory tract infection (URTI) | 44 (15) |
| Otitis media-related^ | 20 (7) |
| Fever | 14 (5) |
| Cough | 7 (2) |
| Other | 60 (21) |
| *Acute bronchiolitis/bronchitis* | *7 (2)* |
| *Presumed gastro-enteritis infection* | *6 (2)* |
| *Other viral disease* | *5 (2)* |
| *Varicella* | *4 (1)* |
| *Dermatitis/ atopic eczema* | *4* |
| *Asthma* | *3* |
| *Acute tonsillitis* | *2* |
| *Acute laryngitis/tracheitis* | *2* |
| *Influenza* | *2* |
| *Wheezing* | *2* |
| *Trauma/injury not otherwise specified* | *2* |
| *Head injury other* | *2* |
| *Observe/Educate/Advice/Diet* | *2* |
| *Other perinatal morbidity* | *1 (0.4)* |
| *Other viral exanthema* | *1* |
| *No disease* | *1* |
| *Vomiting* | *1* |
| *Diarrhoea* | *1* |
| *Oesophageal disease* | *1* |
| *Eustachian tube dysfunction* | *1* |
| *Infections of the musculoskeletal system* | *1* |
| *Congenital musculoskeletal anomaly* | *1* |
| *Shortness of breath/dyspnoea* | *1* |
| *Throat symptom/complaint* | *1* |
| *Naevus/mole* | *1* |
| *Impetigo* | *1* |
| *Loss of appetite* | *1* |
| *Dehydration* | *1* |
| *Cystitis/other urinary infection* | *1* |
| *Sepsis/puerperal infection* | *1* |
